# An open-label cluster-randomised trial on TB preventive therapy for children

**DOI:** 10.5588/ijtldopen.24.0467

**Published:** 2025-03-12

**Authors:** G. Lemvik, L. Larsson, F. Rudolf, J.E. Vejrum, M. Sodemann, V.F. Gomes, C. Wejse

**Affiliations:** ^1^ Bandim Health Project, Bissau, Guinea-Bissau;; ^2^ Bandim Health Project, University of Southern Denmark, Copenhagen, Denmark;; ^3^ Department of Infectious Diseases, Aarhus University Hospital, Aarhus, Denmark;; ^4^ Department of Paediatrics and Adolescent Medicine, Aarhus University Hospital, Aarhus, Denmark;; ^5^ Department of Infectious Diseases, Odense University Hospital, Odense, Denmark;; ^6^ Center for Global Health (GLOHAU), Department of Public Health, Aarhus University, Aarhus, Denmark.

**Keywords:** isoniazid preventive therapy, barriers of adherence, prevention and control, tuberculosis, children

## Abstract

**BACKGROUND:**

In a study on 9 months of isoniazid preventive therapy (IPT) in children in Guinea-Bissau, 76% of children exposed to TB at home completed 6 months of IPT. We aimed to test whether 4 months of rifampicin and isoniazid (RH) would improve adherence compared to 9 months of isoniazid (INH).

**METHODS:**

We conducted an open-label cluster-randomised superiority study in children aged <15 years living with a TB case. Children were randomised by house to receive 4 months of RH or 9 months of INH. RH was given as a fixed-combination pill. The primary outcome was adherence, defined as taking >80% of prescribed dosages per month, assessed by pill count. Our aim was 3 months of RH or 6 months of INH.

**RESULTS:**

A total of 752 children from 223 houses were included, 354 in the INH group and 398 in the RH group. Overall, 57% of the children took >80% of the prescribed pills. In the INH group, 68% completed 6 months of therapy, while 61% of the RH group completed 3 months (OR 1.32, 95% CI 0.90–1.95). The main reason for non-adherence in both groups was travel or relocation, accounting for 50% of missed doses.

**CONCLUSION:**

The shorter preventive therapy of 4 months of RH did not improve adherence in children in Guinea-Bissau. Travelling was the primary reason for non-adherence.

Isoniazid (INH) has been used to treat TB infection (TBI) since the mid-twentieth century. Large randomised trials have found INH to be safe and prevent about 75% of TB cases.^1^ Serious adverse events due to INH are rarely seen in children.^2–5^ Household contacts aged <5 years are at a high risk of contracting TB and should be treated for TBI.^6,7^

Adherence is important for the efficacy of isoniazid preventive therapy (IPT).^8^ A study from South Africa found that only 20% of children completed >5 months of unsupervised IPT.^9^ The length of therapy may pose a challenge to adherence, leading to research on short-term regimens.^2,10–13^ Recent studies have compared IPT with shorter rifamycin-containing regimens, finding better adherence to these shorter treatment regimens,^3–5,14^ reviews also highlight better completion of the shorter treatment options.^15,16^ To date, there has not been a large randomised single-centre study from a country with high TB incidence that compares IPT to short-term regimens in children.

The WHO recently updated its guidelines to include short-term therapies for children. Currently, recommendations are either daily INH for at least 6 months, daily INH combined with rifampicin (RIF) for 3 months or weekly INH and rifapentin for 3 months.^7^ Although TB preventive therapy (TPT) is recommended, only 8–20% of children with TBI receive it,^17,18^ in resource-limited settings, the percentages are at the low end of this scale.

In Guinea-Bissau, an observational study reported that 76% of the children completed 6 months of IPT.^19^ It was also shown in Guinea-Bissau that children living with TB cases had increased all-cause mortality,^20^ but when given IPT, the mortality resembled that of the background population.^21^

Our objective was to investigate whether a shorter course of treatment could further improve adherence in the mobile population of Bissau. We used a cluster-randomised, open-label study design to compare adherence of 4 months of RIF and INH (RH) with 9 months of INH.

The cluster-randomised design was chosen to make it easier on the parents and prevent the possibility that the parent/guardian would switch the medication if they thought the children would benefit more from one of the two therapies.

## METHODS

The study was conducted from 18 January 2011 to 20 August 2013 in the six areas comprising the Bandim Health Project (BHP) study area in Bissau, the capital of Guinea-Bissau. BHP is a Health and Demographic Surveillance System (HDSS) that monitors a population of about 100,000 individuals through regular censuses that track birth, death and migration.

### TB surveillance

Since 1996, all TB cases in the study area have been registered by a TB surveillance system. The TB incidence was 279/100,000 person-years of observation at the time of this study.^22^

### Screening of children’s contacts

The census was updated in the houses of new TB cases. All children <15 years of age living in these houses were invited to undergo screening at the health centre, where they were interviewed and had a tuberculin skin test (TST) placed on the left underarm. A follow-up visit was conducted at their home 48–72 h later, measuring the TST response. An induration >5 mm was considered positive for TBI.

We informed the parents/guardians about the study twice, when visiting the house, inviting the children for screening, and again at the health centre.

### Inclusion/exclusion criteria

Children <5 years of age were included at the health centre screening visit if they did not show signs and symptoms of TB disease. Their TST results did not affect their inclusion in the study. Children 5–14 years of age were included based on a positive TST reading at home, provided they did not show signs and symptoms of TB disease. Children presenting with fever >14 days, cough >14 days, weight loss, enlarged lymph nodes, gibbous, osseous oedema or joint oedema, were tested for TB disease and HIV by chest X-ray, sputum smear if possible, and an HIV test. If TB and HIV were ruled out, the children were included as long as they met the inclusion criteria.

Exclusion criteria were pregnancy (girls >12 years of age had a pregnancy test), a history of seizures, hepatitis and HIV-positive children receiving highly active antiretroviral therapy – the latter due to the concerns regarding potential interaction between rifamycins and antiretroviral therapy (ART). HIV-positive children would be offered 9INH but were not included in the study.

### Randomisation

The study was open-label cluster-randomised, with randomisation by house. A computer-generated randomisation list was made without stratification or matching. Numbered opaque envelopes were prepared to contain the corresponding folded and stapled randomisation lot. All envelopes from the randomisation list were prepared in a single session. The houses with TB cases were consecutively assigned envelopes as they were registered. When the first child from a given house was included, the relevant envelope was opened, revealing the treatment for that cluster. The person who prepared the envelopes was not involved in the inclusion of the children. The randomisation list was sent to the sponsor of the study after the preparation of the envelopes to ensure that it was kept at two different locations and to maintain a means of verifying whether the randomisation was conducted as planned.

### Treatment groups

The children receiving INH were given a dosage of 10–15 mg/kg per day, with a maximum dosage of 300 mg. Initially, the plan was to use only 100 mg pills. However, midway through the study, a delay in the delivery of a new batch of 100 mg pills necessitated a switch to 300 mg INH pills. This change was documented, and the dosage change was included as a risk factor in the analysis for non-completion.

Children receiving 4 months of RH were given fixed-combination pills containing 150 mg RIF and 75 mg INH, with a maximum dosage of 600 mg RIF and 300 mg INH. Their dosage was calculated based on the RIF regimen, with 15–20 mg/kg/day, which resulted in a dose of 7.5–10 mg/kg/day of INH. All children received a daily supplement of 25 mg pyridoxine.

### Adherence and follow-up

We dispensed pills for 2 weeks at a time. Every 2 weeks, research assistants visited the children’s homes to provide the subsequent supply of medication and document adverse events, consultations and hospitalisations. If symptoms of liver damage occurred, liver transaminases were measured; if they exceeded three times the upper normal value, treatment was discontinued.

Adherence was measured by pill count, and the dates and reasons for non-adherence were noted. In some cases, parents or guardians were unable to find the pillbox when assistants came to collect it. In this event, we regarded all these dosages as missed, and labelled them ‘unknown’. The last follow-up was 2 years after inclusion when data on TB morbidity and all-cause mortality were collected.

### Sample size

We expected the adherence of 6 months in total of INH to be 75% and the adherence to 4RH (at least 3 months RH with 80% pills taken) to be 85%. With a mean cluster size of 4 children per cluster and an intra-cluster correlation coefficient (ICC) of 0.1, we would need 161 clusters, a total of 644 children. Expecting a loss to follow-up of 25%, we planned to include 220 clusters, equal to 880 children.

### Statistics

Data were registered in standard questionnaires, entered using dBase V Software (Borland International Inc, Austin, TX, USA), and checked for errors. Results were analysed at the individual level, adjusting for the clusters. Adherence was calculated as the percentage of dosages taken in a given month, and adherence >80% was defined as completion of a month. Completing 3 months of RH or 6 months of INH was defined as completing the regimen. We present overall adherence, month-by-month adherence, total months of adherence, and consecutive months of adherence to both therapies. We compared adherence in the two randomisation groups by odds ratio (OR) with 95% confidence intervals (CIs). The significance level was set to 5%, and we used robust standard errors, adjusting for the clusters.

Risk factors were analysed for non-completion of total and consecutive treatment months. In the multivariate analysis, we analysed the previously mentioned INH dosage change and adverse events as well as age group, residential area and proximity to the index case, which were identified as significant risk factors in the previous study.^19^ The risk factor analysis reported ORs with 95% CIs, and we used robust standard errors, adjusting for the clusters.

Data were analysed according to the intention-to-treat principle. One cluster (one child) was randomised to RH but got INH.

### Ethics

This study was registered at the Pan-African Clinical Trials Registry at PACTR201101000273931. The study was approved by the ethical committees of both Guinea-Bissau (039/CNES/2010) and Denmark (CVK, 1007108). The parents/guardians were informed verbally in Creole, and written information was provided in Portuguese. Informed consent was given by signature by the parents/guardians at the screening or by fingerprint if illiterate. The trial was monitored by a good clinical practice (GCP) trained monitor and a Data Safety Monitoring Board (DSMB) received 6-month reports on adverse events, consultations, hospitalisations, deaths, and loss to follow-up in addition to the GCP reports.

## RESULTS

**Figure 1.**
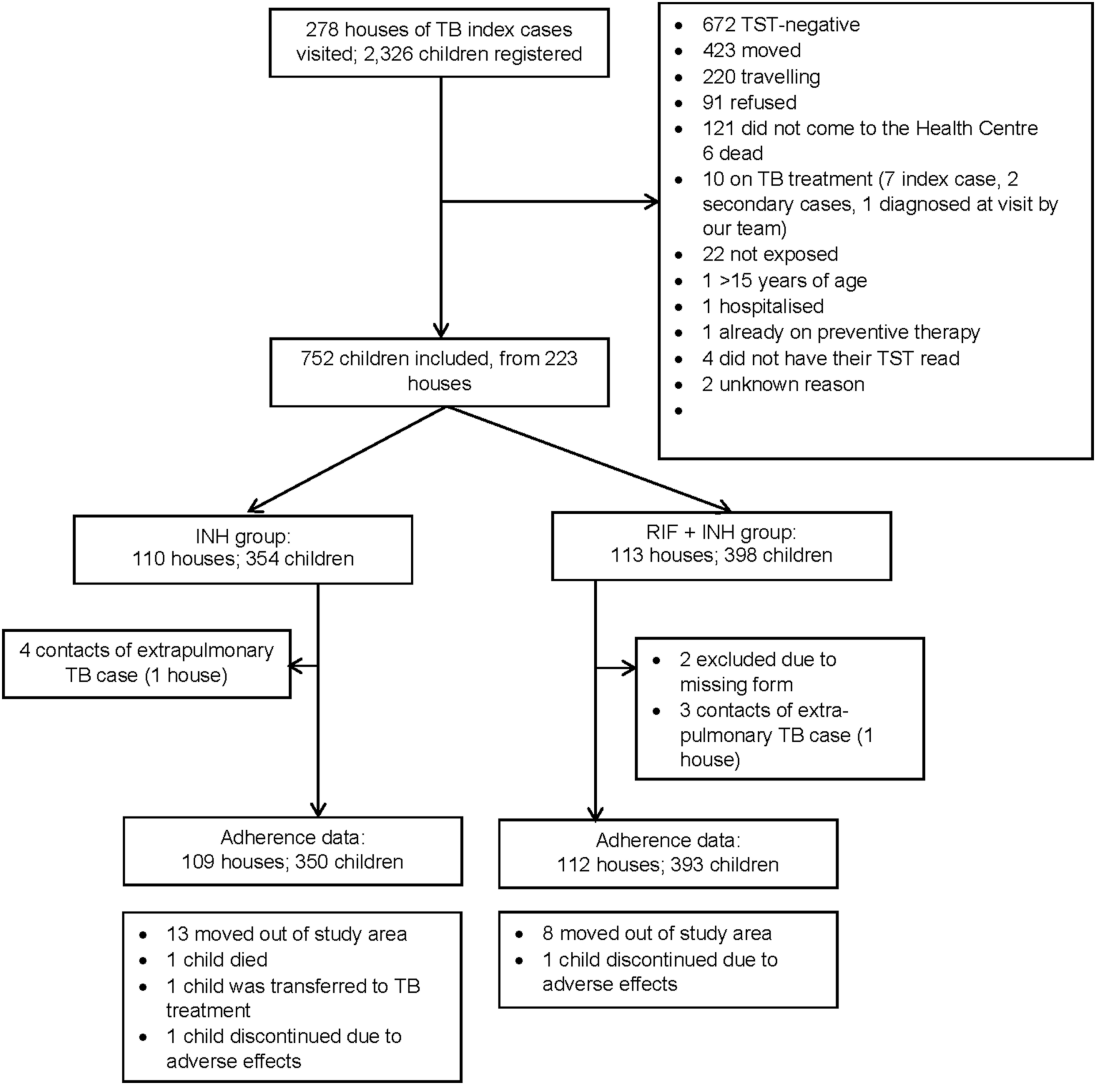
Inclusion flowchart. TST = tuberculin skin test; INH = isoniazid; RIF = rifampicin.

Between January 2011 and November 2012, we visited the houses of 278 TB index cases; 2,326 children were registered as living in these houses, and some had moved, died or were travelling (Figure 1). We screened 1,432 children at the health centre, of which 675 tested TST-negative, and four did not have their TST read. Inclusion was stopped when we reached the targeted number of clusters.

We included 752 children from 223 houses, 354 children from 110 houses assigned to the INH group, and 398 children from 113 houses assigned to the RH group. Two follow-up forms were lost during the trial, leading to the exclusion of these two children in the RH group. Additionally, two clusters were excluded because the index case had extra-pulmonary TB: one in the INH group (four children) and one in the RH group (three children). Adherence follow-up ended in August 2013, and 221 houses entered the adherence analysis: 109 houses with 350 children in the INH group and 112 houses with 393 children in the RH group. Background factors are presented in Table 1.

**Table 1.** Background information of participants at individual and cluster level.

	9 months isoniazid (109 clusters, 350 children) *n* (%)	4 months rifampicin+isoniaizid (112 clusters, 393 children) *n* (%)
Individual level		
Boys	163 (47)	182 (46)
Age, years, mean ± SD	4.6 ± 0.2	4.6 ± 0.2
Level of proximity to index case:		
Same bed	14 (4)	26 (6.5)
Same room	10 (3)	20 (5)
Same household	59 (17)	75 (19)
Same house	267 (76)	270 (69)
Other house	0 (0)	2 (0·5)
Ethnicity		
Pepel	117 (33)	140 (36)
Manjaco	38 (11)	52 (13)
Mancanha	25 (7)	41 (10)
Balanta	19 (5)	34 (9)
Fula	45 (13)	52 (13)
Mandinga	37 (11)	25 (6)
Other	69 (20)	49 (13)
Area of residence		
Bandim 1	126 (36)	142 (36)
Bandim 2	43 (12)	65 (16)
Belem	50 (14)	50 (13)
Mindara	31 (9)	24 (6)
Cuntum 1	47 (14)	69 (18)
Cuntum 2	53 (15)	43 (11)
Cluster level, mean ± SD		
Number of children living/cluster	8.3 ± 0.2	8.4 ± 0.2
Number of children included/cluster	3.2 ± 0.2	3.5 ± 0.2

SD = standard deviation.

Forty-three of the screened children showed symptoms of TB at screening and were checked by chest X-ray, sputum smear if possible, HIV test and clinical follow-up; one of them was diagnosed with TB. All were HIV-negative.

During the study, one child in the INH group died, with a diagnosis of typhoid fever. There were no signs of jaundice or liver failure, leading to the conclusion that the death was probably not related to the study medication. No additional deaths were reported among the included children during the two-year follow-up period. Five screened, non-included children died during this time. (OR 0.24, 95% CI 0.03–2.10)

### Adherence

**Figure 2.**
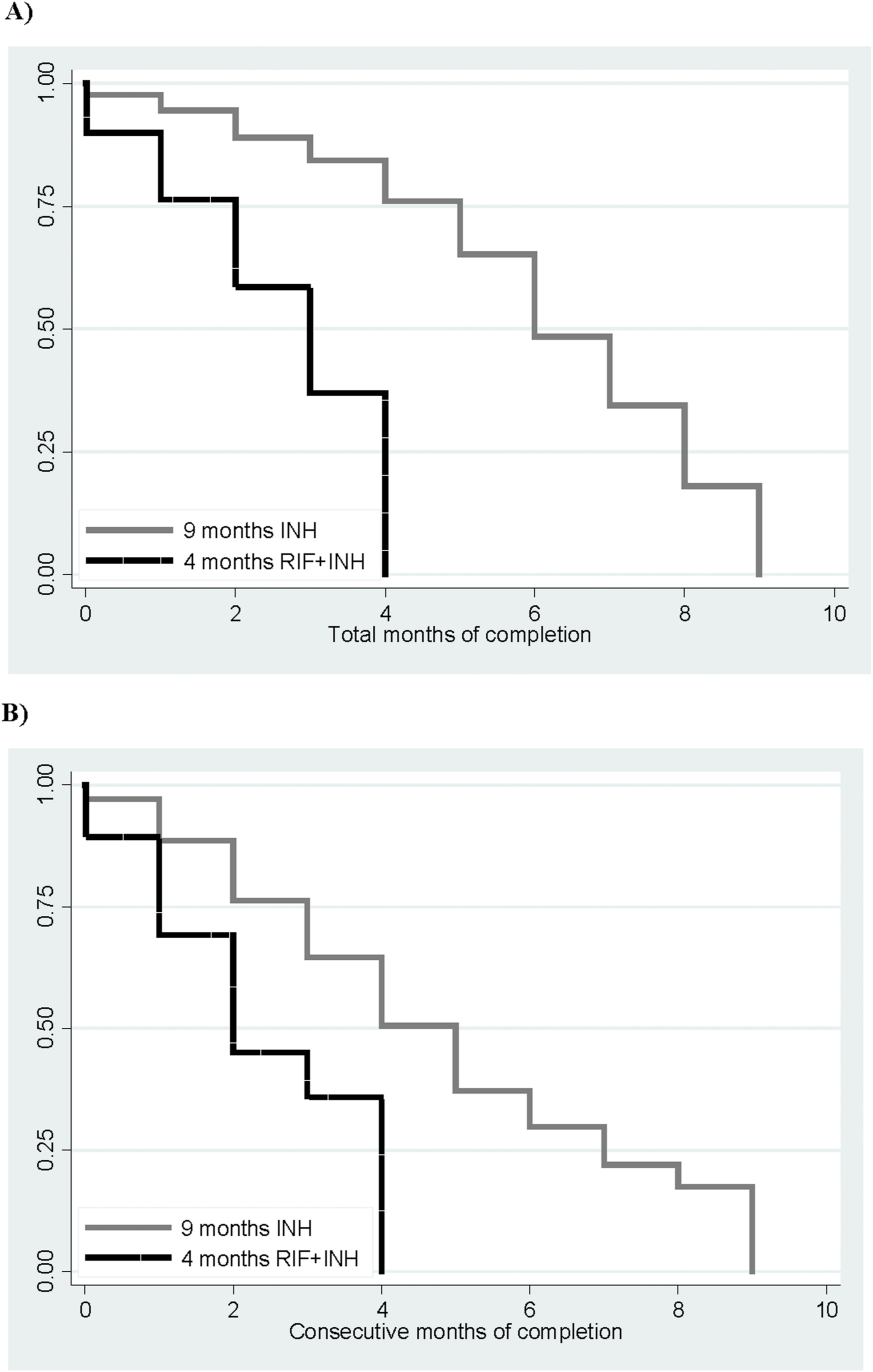
**A)** Kaplan-Meier plot of total months of adherence to therapy for the two groups. Percentages refer to total number of included individuals. **B)** Kaplan-Meier plot of consecutive months of adherence to therapy for the two groups. Percentages refer to total number of included individuals. INH = isoniazid; RIF = rifampicin.

Overall, the children took a mean of 77% (95% CI 0.76–0.79) of the prescribed pills; 57% took >80% of the prescribed pills, 56% in the INH group and 58% in the RH group. Treatment completion is presented as the total number of completed months and the consecutive number of completed months (Table 2A and B, Figure 2A and B). Month-by-month adherence is also shown in Table 2.

**Table 2.** **A)** Completion of rifampicin+isoniazid therapy by month.

	1 month (*n* = 393)^*^ *n* (%)	2 months (*n* = 377)^*^ *n* (%)	3 months^†^ (*n* = 367)^*^ *n* (%)	4 months (*n* = 356)^*^ *n* (%)
Total completed months (>80%)	351 (89)	296 (79)	223 (61)^†^	141 (40)
Consecutive completed months (>80%)	351 (89)	272 (72)	177 (48)^†^	141 (40)
Month-by-month	257 (65)	245 (65)	239 (65)^†^	270 (76)

Sixty-eight per cent of the INH group completed their therapy, compared to 61% of the RH group (OR 1.32, 95% CI 0.90–1.95), and the ICC was 0.24 (95% CI 0.16–0.32). When completing consecutive months of treatment, 40% of the INH group completed versus 48% of the RH group (OR 0.72, 95% CI 0.47–1.10), ICC (OR 0.30, 95% CI 0.22–0.39). Month-by-month adherence for the RH group was stable at 65%, peaking at 76% in the final month. In the INH group, adherence began at 69%, declined to 61% by month 4, and rose above 70% in the last 4 months.

### Reasons for missed dosages

Overall, 23% of the prescribed dosages were not taken (Table 3). The missing dosages were evenly distributed between the two groups; a higher percentage of children in the INH group moved (11% versus 5%), whereas more children in the RH group forgot their dosages (34% versus 27%).

**Table 3.** Reasons for missing dosages.

	Isoniazid *n/N* (%)	Rifampicin + isoniazid *n/N* (%)	Total *n/N* (%)
Total missing dosages	22,633/95,900 (24)	11,197/48,476 (23)	33,830/144,376 (23)
Moved	2,383 (11)	598 (5)	2,981 (9)
Travelling	9,545 (42)	4,401 (39)	13,946 (41)
Forgotten	6,103 (27)	3,798 (34)	9,901 (29)
Lost pills	18 (0.1)	0 (0)	18 (0.05)
Refused	295 (1)	278 (2)	573 (2)
Adverse effects	27 (0.1)	54 (0.5)	81 (0.2)
Failure of distribution	92 (0.4)	65 (0.6)	157 (0.5)
Other	290 (1)	69 (0.6)	359 (1)
Unknown (pill box not found)	3,880 (17)	1,934 (17)	5,814 (17)

### Risk factors for non-completion (Table 4)

Children aged 5–14 years had a lower risk of non-completion than younger children, with ORs of 0.40 (95% CI 0.27–0.59) for total months and 0.60 (95% CI 0.42–0.86) for consecutive months. In the total months’ analysis, children living in the Cuntum 2 district had a significantly lower risk of non-completion (OR 0.42, 95% CI 0.23–0.76); this was not seen in the consecutive months’ analysis. Children who presented with mild symptoms at home had a lower risk of non-completion, both for the total OR of 0.45 (95% CI 0.30–0.69) and for the consecutive months analysis (OR 0.56, 95% CI 0.37–0.84). If the symptoms warranted consultation or hospitalisation, the lower risk of non-completion disappeared. Children whose mothers had >7 years of schooling had a significantly lower risk of non-completion for total and consecutive months (OR 0.41, 95% CI 0.23–0.72 and OR 0.55, 95% CI 0.31–0.99, respectively). The dosage change in the INH group was a significant risk factor for non-completion of consecutive months (OR 1.97, 95% CI 1.07–3.64), which was not seen in the total months analysis.

**Table 4.** Risk factors for non-completion of total/consecutive months of INH/RIF+INH: univariate and multivariate analysis.

	Non-completion of total months of therapy			Non-completion of consecutive months of therapy		
	cOR (95% CI)	aOR (95% CI)	*p*-value	cOR (95% CI)	aOR (95% CI)	*p*-value
Duration of treatment						
9 months INH	1	1		1	1	
4 months RIF+INH	1.32 (0.90–1.95)	1.22 (0.72–2.04)	0.46	0.72 (0.47–1.10)	0.91 (0.55–1.50)	0.70
Sex						
Male	1			1		
Female	0.94 (0.70–1.27)			0.92 (0.68–1.26)		
Age, years						
<5	1	1		1	1	
5–15	0.52 (0.37–0.73)	0.40 (0.27–0.59)	<0.01	0.71 (0.52–0.98)	0.60 (0.42–0.86)	<0.01
INH group only						
Same dosage	1	1		1	1	
Changed dosage	1.13 (0.66–1.95)	1.07 (0.62–1.84)	0.82	1.79 (0.98–3.26)	1.97 (1.07–3.64)	0.03
Residence						
Same house	1	1		1	1	
Same household	0.90 (0.55–1.47)	0.97 (0.60–1.58)	0.90	0.80 (0.48–1.34)	0.84 (0.49–1.43)	0.52
Same room	1.46 (0.66–3.23)	1.79 (0.76–4.20)	0.18	1.15 (0.45–2.98)	1.31 (0.54–3.18)	0.55
Same bed	0.79 (0.38–1.66)	0.82 (0.36–1.87)	0.63	0.82 (0.40–1.67)	0.88 (0.40–1.96)	0.76
Ethnicity						
Pepel	1			1		
Manjaco	0.70 (0.40–1.23)			0.87 (0.47–1.63)		
Mancanha	0.81 (0.40–1.64)			0.97 (0.48–1.93)		
Balanta	0.53 (0.23–1.19)			0.60 (0.27–1.31)		
Fula	1.31 (0.73–2.34)			1.04 (0.59–1.84)		
Mandinga	0.90 (0.45–1.83)			1.00 (0.54–1.81)		
Other	0.79 (0.47–1.34)			1.01 (0.54–1.90)		
Area of residence						
Bandim 1	1	1		1	1	
Bandim 2	1.00 (0.53–1.89)	1.00 (0.50–2.01)	0.99	0.88 (0.46–1.70)	0.96 (0.48–1.91)	0.90
Belem	1.70 (0.95–3.02)	1.55 (0.83–2.89)	0.17	1.91 (0.92–3.98)	1.82 (0.84–3.97)	0.13
Mindara	0.73 (0.32–1.68)	0.84 (0.38–1.86)	0.67	0.73 (0.33–1.62)	0.73 (0.32–1.68)	0.46
Cuntum 1	1.27 (0.69–2.32)	0.98 (0.51–1.86)	0.95	1.12 (0.56–2.21)	1.03 (0.51–2.05)	0.94
Cuntum 2	0.50 (0.27–0.93)	0.42 (0.23–0.76)	<0.01	0.68 (0.37–1.23)	0.65 (0.37–1.16)	0.15
Adverse events						
No adverse events	1			1		
Adverse events	0.63 (0.44–0.90)			0.76 (0.53–1.08)		
Symptoms at home	0.52 (0.34–0.78)	0.45 (0.30–0.69)	<0.01	0.67 (0.46–0.97)	0.56 (0.37–0.84)	<0.01
Consultation	0.70 (0.39–1.25)	0.61 (0.34–1.12)	0.11	0.76 (0.43–1.34)	0.61 (0.34–1.11)	0.10
Hospitalisation	1.76 (0.67–4.57)	1.97 (0.73–5.34)	0.18	2.39 (0.63–9.07)	2.75 (0.62–12.18)	0.18
Mother’s schooling						
No schooling	1	1		1	1	
1–6 years of schooling	0.82 (0.47–1.40)	0.72 (0.41–1.24)	0.23	0.84 (0.46–1.52)	0.83 (0.45–1.52)	0.55
>7 years of schooling	0.51 (0.30–0.88)	0.41 (0.23–0.72)	<0.01	0.57 (0.33–0.98)	0.55 (0.31–0.99)	0.05
No info on schooling	0.64 (0.34–1.20)	0.73 (0.38–1.39)	0.34	0.84 (0.42–1.69)	1.00 (0.48–2.06)	1.00

INH = isoniazid; RIF = rifampicin; cOR = crude odds ratio; CI = confidence interval; aOR = adjusted OR.

### Loss to follow-up

Twenty-one children were lost to follow-up because they moved, 13 in the INH group and 8 in the RH group. Sixty-four children travelled during the study period and had not returned at the last adherence follow-up, 26 in the INH group and 38 in the RH group.

### Tuberculosis

One child in the INH group was diagnosed with TB 12 weeks after enrolment. Additionally, one non-included child contracted TB during the 2-year follow-up.

### Adverse events

A total of 258 children reported symptoms during the study: 163 in the INH group (∼18 per month) and 95 in the RH group (∼24 per month). The most commonly reported symptoms were fever and cough. Seventy-nine of these children went for 101 consultations: 65 consultations in the INH group (∼7 per month) and 36 in the RH group (∼9 per month). A total of 19 children were admitted to the hospital: 14 in the INH group (∼1.6 per month) and 5 in the RH group (∼1.3 per month). We have not reported the reddening of urine as an adverse event, as all children on RH experienced this well-known and harmless side effect.

### Treatment discontinuation

Thirty-two children had transaminase levels measured due to symptoms of liver disease. Of these, one child had enzymes>3 times the upper normal limit, prompting the discontinuation of RH treatment. The liver enzymes returned to normal values within two months of discontinuation. One child in the INH group experienced convulsions, leading to discontinuation of their treatment.

## DISCUSSION

Although our data are not recent, this is a large randomised controlled trial from a high-incidence country, and we believe the results are still relevant. The public health scenario in Guinea-Bissau has not changed significantly since the study was conducted. We included the planned number of clusters; however, fewer children were in each cluster than anticipated. We had initially planned for a loss-to-follow-up of 25%, but the actual loss was much lower, at 11%. As a result, the sample size remained adequate. We initially expected the completion of RH to surpass that of INH but found slightly higher adherence in the INH group – although this was not statistically significant. While completion rates for consecutive months were higher for the RH regimen than the INH regimen, this difference lacked statistical significance. These findings contradict the results from other published randomised^3–5^ and observational^14^ studies. Our study is the only large randomised study from a single high-incidence country. While its results cannot be generalised globally, they provide valuable insight into a West African context. We did not find better completion rates for the short-term regimen in our setting. Both regimens achieved adherence rates >60%, yet the rates were lower than those in a previous observational study.^19^ Higher adherence has also been seen in other parts of Africa – 86% in a study in Benin,^23^ and 92% in Ethiopia,^24^ both studies implemented IPT in an already well-functioning TB programme,^23,24^ while an observational study in India showed a low adherence of 23%, and highlighted the challenges in national programme implementation.^25^ A recent cluster randomised controlled trial from Cameroon and Uganda compared a decentralised approach at the household level with the facility-based standard of care and found that the decentralised approach significantly improves the initiation and completion of TPT among child contacts.^26^

Travelling or moving were the most prominent reasons for missed dosages in both treatment groups. As our study population is very mobile, this was an anticipated issue for preventive therapy in this context, although we expected it to be a minor concern in the shorter treatment group.

The second most frequent reason for missing dosages was forgetting to take the pills. This raises concern, as our staff visited the children every two weeks, reminding them to take the pills. A qualitative study from Indonesia identified a lack of knowledge about TB and IPT as significant barriers to adherence;^27^ a study from Ethiopia suggested the same.^28^ Despite the thorough training of our staff and detailed information provided to participants, the understanding may have been sub-optimal. The level of schooling for mothers increased the completion of therapy for the total and consecutive months. This finding implies that a higher level of maternal education may enhance understanding of the complexity of preventive therapy for healthy children. A review identified parental risk perception and gaps in healthcare worker (HCW) knowledge as barriers to IPT implementation.^29^ Health education for TPT candidates has recently been recognised as a clinical TB prevention standard.^30^ Training HCWs to effectively manage TB in children has increased TB case finding and IPT uptake in Uganda.^31^ In both arms of our study, adherence to the therapy increased during the final months. While we cannot fully explain this trend, it is possible that families became more familiar with the therapy as it progressed, or they may have been motivated to make a final effort to take the medication, knowing that the therapy was nearing completion.

Of the missed dosages, 17% were attributed to the unavailable pillbox. In these instances, the field assistants registered not receiving the pillbox and asked the parent/guardian if the children had taken the pills. In every case, the parent/guardian reported that the child had taken all prescribed dosages; this information did not differ between children who usually took all and children who often forgot their dosages. We categorised these as missed dosages, and adherence may be underestimated. If we had considered the information given by the parents/guardians as correct, we would, on the other hand, have overestimated adherence. The previous observational study from Guinea-Bissau did not have measures to detect this problem, so their adherence may be overestimated.

Previously, in our setting, older age was related to higher completion rates of consecutive therapy, while sleeping in the same bed as the index case was a risk factor for non-completion.^19^ We also found older age related to higher completion in this study but did not find the proximity of the index case to be related to adherence. In the previous study, living in the district of Cuntum 2 was related to higher completion of consecutive therapy, while in our study, this was significantly related to completion of total months of treatment but not to completion of consecutive months. This may be a chance finding. However, we have not analysed socio-economic differences between districts, which may explain the differences in both studies.

Completion of therapy was higher if the child had any symptoms at home that did not warrant a consultation. An explanation for this observation could be that having any symptom at home would make the parent/guardian more conscious of the child’s well-being and thus increase awareness of taking medication. We experienced one death in our population of 752 children, which was probably not related to the INH. During the two-year follow-up, this was the only death among the included children, while five children screened but not included died during the same period. This mortality rate is very low for a country like Guinea-Bissau and is comparable to rates observed in the previous IPT study.^21^ Treatment was discontinued in two children due to adverse effects; both children were well after the discontinuation. Hospitalisation was defined as a serious adverse event, and 19 children were hospitalised. None of these hospitalisations were found to be related to the study drugs. Thus, the overall rate of adverse effects from the drugs was low, supporting the conclusion that both INH and RH are safe for use in children, a finding also corroborated by other studies.^4,16,32^

### Limitations

We measured adherence through pill count, which may lead to overestimation, as this method does not confirm that the dosage was taken. It is possible to measure INH concentration in urine,^33^ but this only provides a 24-hour picture of adherence, and the method is not currently available or feasible in Bissau. Directly observed therapy (DOT) would be the ideal approach to measure adherence, but this is difficult to maintain for preventive therapy of healthy children for several months. Unfortunately, we had to modify dosages for some INH children during the study due to supply issues. We included the dosage change in the risk factor analysis, and it was a significant risk factor for non-completion of consecutive months but not for total months. This would likely not have made any difference with DOT, but when a child/parent/guardian has to change a routine, it may very well affect adherence.

In conclusion, a shorter preventive therapy of 4 months of RH did not improve adherence compared with 9 months of INH in Guinean children exposed to TB at home, although both regimens were feasible in our setting. Moving or travelling accounted for 50 % of the missed dosages. Both drugs were safe in children, with only two cases of treatment discontinuation due to adverse reactions.

## References

[1] Ferebee SH, The use of chemotherapy as a prophylactic measure in tuberculosis. Ann N Y Acad Sci. 1963;106:151–156. 13944724 10.1111/j.1749-6632.1963.tb16633.x

[2] Ormerod LP. Rifampicin and isoniazid prophylactic chemotherapy for tuberculosis. Arch Dis Child. 1998;78(2):169–171. 9579163 10.1136/adc.78.2.169 PMC1717463

[3] Spyridis NP, The effectiveness of a 9-month regimen of isoniazid alone versus 3- and 4-month regimens of isoniazid plus rifampin for treatment of latent tuberculosis infection in children: results of an 11-year randomised study. Clin Infect Dis. 2007;45(6):715–722. 17712755 10.1086/520983

[4] Diallo T, Safety and side effects of rifampin versus isoniazid in children. N Engl J Med. 2018;379(5):454–463. 30067928 10.1056/NEJMoa1714284

[5] Villarino ME, Treatment for preventing tuberculosis in children and adolescents: a randomised clinical trial of a 3-month, 12-dose regimen of a combination of rifapentine and isoniazid. JAMA Pediatr. 2015;169(3):247–255. 25580725 10.1001/jamapediatrics.2014.3158 PMC6624831

[6] Nair N. Childhood tuberculosis: public health and contact tracing. Paediatr Respir Rev. 2001;2(2):97–102. 12531055 10.1053/prrv.2000.0116

[7] World Health Organization. WHO consolidated guidelines on tuberculosis: Module 1: prevention – infection prevention and control. WHO Guidelines Approved by the Guidelines Review Committee. Geneva, Switzerland: WHO, 2022.

[8] International Union Against Tuberculosis Committee on Prophylaxis. Efficacy of various durations of isoniazid preventive therapy for tuberculosis: five years of follow-up in the IUAT trial. Bull World Health Organ. 1982;60(4):555–564. 6754120 PMC2536088

[9] Marais BJ, Adherence to isoniazid preventive chemotherapy: a prospective community-based study. Arch Dis Child. 2006;91(9):762–765. 16737993 10.1136/adc.2006.097220 PMC2082929

[10] Hong Kong Chest Service/Tuberculosis Research Centre, Madras/British Medical Research Council. A double-blind placebo-controlled clinical trial of three antituberculosis chemoprophylaxis regimens in patients with silicosis in Hong Kong. Am Rev Respir Dis. 1992;145(1):36–41. 1731596 10.1164/ajrccm/145.1.36

[11] Martinez Alfaro E, [Compliance, tolerance and effectiveness of a short chemoprophylaxis regimen for the treatment of tuberculosis]. Med Clin (Barc). 1998;111(11):401–404. [Spanish] 9834911

[12] Chan PC, Latent tuberculosis infection treatment for prison inmates: a randomised controlled trial. Int J Tuberc Lung Dis. 2012;16(5):633–638. 22410137 10.5588/ijtld.11.0504

[13] Trajman A, Factors associated with treatment adherence in a randomised trial of latent tuberculosis infection treatment. Int J Tuberc Lung Dis. 2010;14(5):551–559. 20392347

[14] Cruz AT, Safety and completion of a 4-month course of rifampicin for latent tuberculous infection in children. Int J Tuberc Lung Dis. 2014;18(9):1057–1061. 25189552 10.5588/ijtld.14.0286

[15] Pease C, Efficacy and completion rates of rifapentine and isoniazid (3HP) compared to other treatment regimens for latent tuberculosis infection: a systematic review with network meta-analyses. BMC Infect Dis. 2017;17(1):265. 28399802 10.1186/s12879-017-2377-x PMC5387294

[16] Assefa Y, 3-month daily rifampicin and isoniazid compared to 6- or 9-month isoniazid for treating latent tuberculosis infection in children and adolescents less than 15 years of age: an updated systematic review. Eur Respir J. 2018;52(1):1800395. 29748305 10.1183/13993003.00395-2018 PMC6095703

[17] van Wyk SS, Operational challenges in managing isoniazid preventive therapy in child contacts: a high-burden setting perspective. BMC Public Health. 2011;11:544. 21740580 10.1186/1471-2458-11-544 PMC3150266

[18] Hamada Y, Prevention of tuberculosis in household members: estimates of children eligible for treatment. Bull World Health Organ. 2019;97(8):534–547D. 31384072 10.2471/BLT.18.218651 PMC6653819

[19] Gomes VF, Adherence to isoniazid preventive therapy in children exposed to tuberculosis: a prospective study from Guinea-Bissau. Int J Tuberc Lung Dis. 2011;15(12):1637–1643. 22118171 10.5588/ijtld.10.0558

[20] Gomes VF, Impact of tuberculosis exposure at home on mortality in children under 5 years of age in Guinea-Bissau. Thorax. 2011;66(2):163–167. 21148136 10.1136/thx.2010.141309

[21] Gomes VF, Impact of isoniazid preventive therapy on mortality among children less than 5 years old following exposure to tuberculosis at home in Guinea-Bissau: a prospective cohort study. BMJ Open. 2013;3(3):e001545. 10.1136/bmjopen-2012-001545 PMC3612806 23535699

[22] Lemvik G, Decline in overall, smear-negative and HIV-positive TB incidence while smear-positive incidence stays stable in Guinea-Bissau 2004–2011. Trop Med Int Health. 2014;19(11):1367–1376. 25145557 10.1111/tmi.12378

[23] Adjobimey M, Implementation of isoniazid preventive therapy in children aged under 5 years exposed to tuberculosis in Benin. Int J Tuberc Lung Dis. 2016;20(8):1055–1059. 27393539 10.5588/ijtld.15.0493

[24] Datiko DG, A community-based isoniazid preventive therapy for the prevention of childhood tuberculosis in Ethiopia. Int J Tuberc Lung Dis. 2017;21(9):1002–1007. 28826449 10.5588/ijtld.16.0471 PMC5566998

[25] Shivaramakrishna HR, Isoniazid preventive treatment in children in two districts of South India: does practice follow policy? Int J Tuberc Lung Dis. 2014;18(8):919–924. 25199005 10.5588/ijtld.14.0072 PMC4589200

[26] Bonnet M, Effectiveness of a community-based approach for the investigation and management of children with household tuberculosis contact in Cameroon and Uganda: a cluster-randomised trial. Lancet Glob Health. 2023;11(12):e1911–e1921. 37918417 10.1016/S2214-109X(23)00430-8

[27] Rutherford ME, Adherence to isoniazid preventive therapy in Indonesian children: a quantitative and qualitative investigation. BMC Res Notes. 2012;5:7. 22221424 10.1186/1756-0500-5-7 PMC3287144

[28] Garie KT, Lack of adherence to isoniazid chemoprophylaxis in children in contact with adults with tuberculosis in Southern Ethiopia. PLoS One. 2011;6(11):e26452. 22069451 10.1371/journal.pone.0026452 PMC3206033

[29] Grace SG. Barriers to the implementation of isoniazid preventive therapy for tuberculosis in children in endemic settings: a review. J Paediatr Child Health. 2019;55(3):278–284. 30604557 10.1111/jpc.14359

[30] Migliori GB, Clinical standards for the diagnosis, treatment and prevention of TB infection. Int J Tuberc Lung Dis. 2022;26(3):190–205. 35197159 10.5588/ijtld.21.0753 PMC8886963

[31] Zawedde-Muyanja S, Decentralisation of child tuberculosis services increases case finding and uptake of preventive therapy in Uganda. Int J Tuberc Lung Dis. 2018;22(11):1314–1321. 30355411 10.5588/ijtld.18.0025 PMC7237826

[32] Zenner D, Treatment of latent tuberculosis infection: an updated network meta-analysis. Ann Intern Med. 2017;167(4):248–255. 28761946 10.7326/M17-0609

[33] Amlabu V, Isoniazid/acetylisoniazid urine concentrations: markers of adherence to isoniazid preventive therapy in children. Int J Tuberc Lung Dis. 2014;18(5):528–530. 24903787 10.5588/ijtld.13.0730 PMC4229494

